# Association between platelet-to-neutrophil ratio and asthma–COPD overlap: a cross-sectional study in China

**DOI:** 10.3389/fmed.2026.1729278

**Published:** 2026-03-18

**Authors:** Lei Yang, TingTing Zeng, Na Li, DePeng Jiang

**Affiliations:** 1 Department of Respiratory Medicine, The Second Affiliated Hospital of Chongqing Medical University, Chongqing, China; 2 Department of Endocrinology, The Second Affiliated Hospital of Chongqing Medical University, Chongqing, China

**Keywords:** asthma, chronic obstructive pulmonary disease, neutrophil counts, overlap syndrome, platelets

## Abstract

**Background:**

The platelet-to-neutrophil ratio (PNR) has emerged as a valuable biomarker that reflects both systemic inflammatory activity and overall nutritional status. Asthma–chronic obstructive pulmonary disease (COPD) overlap (ACO) is a clinical syndrome characterized by persistent airway inflammation and recurrent episodes of respiratory deterioration. Although inflammation represents a common underlying pathological mechanism, the relationship between PNR and the incidence or severity of ACO has not yet been fully clarified. Elucidating this association is therefore of considerable clinical and scientific importance.

**Methods:**

This cross-sectional retrospective study used multivariable logistic regression analyses to examine the association between PNR and ACO, after adjusting for key covariates, including age, sex, body mass index (BMI), hemoglobin, white blood cell count, eosinophil count, creatinine, alanine aminotransferase, aspartate aminotransferase, albumin levels, smoking history, and alcohol consumption. Restricted cubic spline (RCS) models assessed linear and non-linear relationships, while Spearman’s correlation analysis evaluated the strength and direction of associations. In addition, subgroup analyses were performed to investigate potential differences across specific population groups.

**Results:**

A total of 1,025 participants were included in the analysis, with a median age of 61 years; 72.78% of the cohort were male. The study population comprised 685 healthy controls (HCs), 348 individuals with asthma, 372 with chronic obstructive pulmonary disease (COPD), and 340 with asthma–COPD overlap (ACO). Patients with ACO exhibited significantly lower PNR values than HCs (*p* < 0.05). After adjusting for potential confounders, PNR remained independently associated with a reduced risk of ACO (odds ratio [OR] = 0.964, 95% confidence interval [CI]: 0.954–0.975; *p* < 0.0001). RCS analyses confirmed a dose–response relationship between PNR and ACO risk. A non-linear association was identified, with a threshold inflection point at a PNR value of 61.17. Above this threshold, higher PNR levels remained significantly associated with a lower risk of ACO (OR = 0.926, 95% CI: 0.905–0.948; *p* < 0.001). Spearman’s correlation analysis demonstrated a moderate negative correlation between PNR and ACO (*r* = −0.447, *p* < 0.001). Receiver operating characteristic (ROC) analysis yielded an area under the curve (AUC) of 0.774 (95% CI: 0.742–0.806), indicating acceptable discriminatory performance.

**Conclusion:**

This study demonstrates a significant inverse association between PNR and ACO, particularly when PNR values fall below the identified threshold of 61.17. These findings suggest that PNR may serve as a potentially valuable biomarker for assessing ACO risk. Further prospective and validation studies are warranted to confirm its diagnostic performance and clinical applicability.

## 1 Introduction

Chronic obstructive pulmonary disease (COPD) represents one of the most pressing global public health challenges and is projected to increase in prevalence over the coming decades (1). This rising disease burden is largely associated with population aging and ongoing environmental changes. Simulation data from the Global Burden of Disease database indicate that the number of COPD cases among individuals aged 25 years and older is expected to increase by 23%, reaching approximately 600 million by 2050 (2). Asthma also remains a major public health concern worldwide. The Global Asthma Report estimates asthma prevalence rates of 9.1% in children, 11.0% in adolescents, and 6.6% in adults (3). Consistently, a previous study reported that, in 2021, the global prevalence of asthma was approximately 3,340 cases per 100,000 individuals (4). Patients who exhibit overlapping clinical and physiological characteristics of both asthma and COPD are commonly classified as having asthma-COPD overlap (ACO). Prior evidence suggests that approximately 15% of individuals diagnosed with COPD based on spirometric criteria may actually meet diagnostic features consistent with ACO (5). However, substantial heterogeneity in existing ACO definitions continues to complicate accurate diagnosis and optimal clinical management (6).

In recent years, absolute blood cell counts and their derived ratios have attracted increasing attention as readily accessible inflammatory biomarkers in COPD, asthma, and related overlap syndromes (7–9). Platelets play a pivotal role in the pathophysiology of COPD by contributing to thrombotic, inflammatory, and immune processes within the pulmonary microenvironment. A meta-analysis by Zinellu et al. demonstrated that platelet-related biomarkers are associated with both stable COPD—particularly, platelet count and platelet-to-lymphocyte ratio [PLR]—and acute exacerbations of COPD—particularly, PLR (10). In addition, mean platelet volume has been identified as a negative predictor of acute exacerbations in patients with COPD (11). Neutrophils are key effectors of innate immune responses in the respiratory system and can induce inflammatory lung injury across different age groups (12, 13). In COPD, neutrophils may become excessively activated, releasing proinflammatory mediators such as interleukin-8 (IL-8), recruiting additional neutrophils to sites of inflammation, and promoting oxidative stress through the generation of reactive oxygen species (14). The platelet-to-neutrophil ratio (PNR), an emerging composite biomarker that integrates platelet and neutrophil counts, may provide a more comprehensive reflection of thrombosis, inflammation, and their interplay. Previous studies have demonstrated the clinical relevance of PNR across a range of diseases, including lung cancer, diabetic retinopathy, sickle cell anemia, and cerebrovascular disorders, with lower PNR values often indicating a poorer prognosis (15–19). Notably, evidence suggests that PNR, together with the lymphocyte-to-monocyte ratio, may outperform traditional inflammatory indices, such as the neutrophil-to-lymphocyte ratio (NLR), PLR, and systemic immune-inflammation index (SII), in predicting outcomes in ovarian cancer (20). Similarly, another study reported that PNR was a superior predictor of carotid atherosclerosis compared with NLR, PLR, and SII (21).

Despite its emerging clinical relevance, evidence examining the association between PNR and ACO remains limited. This knowledge gap underscores the need for further investigation. To address this limitation, the present study employed a cross-sectional analytical design to systematically evaluate the relationship between PNR and ACO, thereby providing novel evidence in an area where data are currently scare.

## 2 Materials and methods

### 2.1 Study design and population

**Figure 1:**
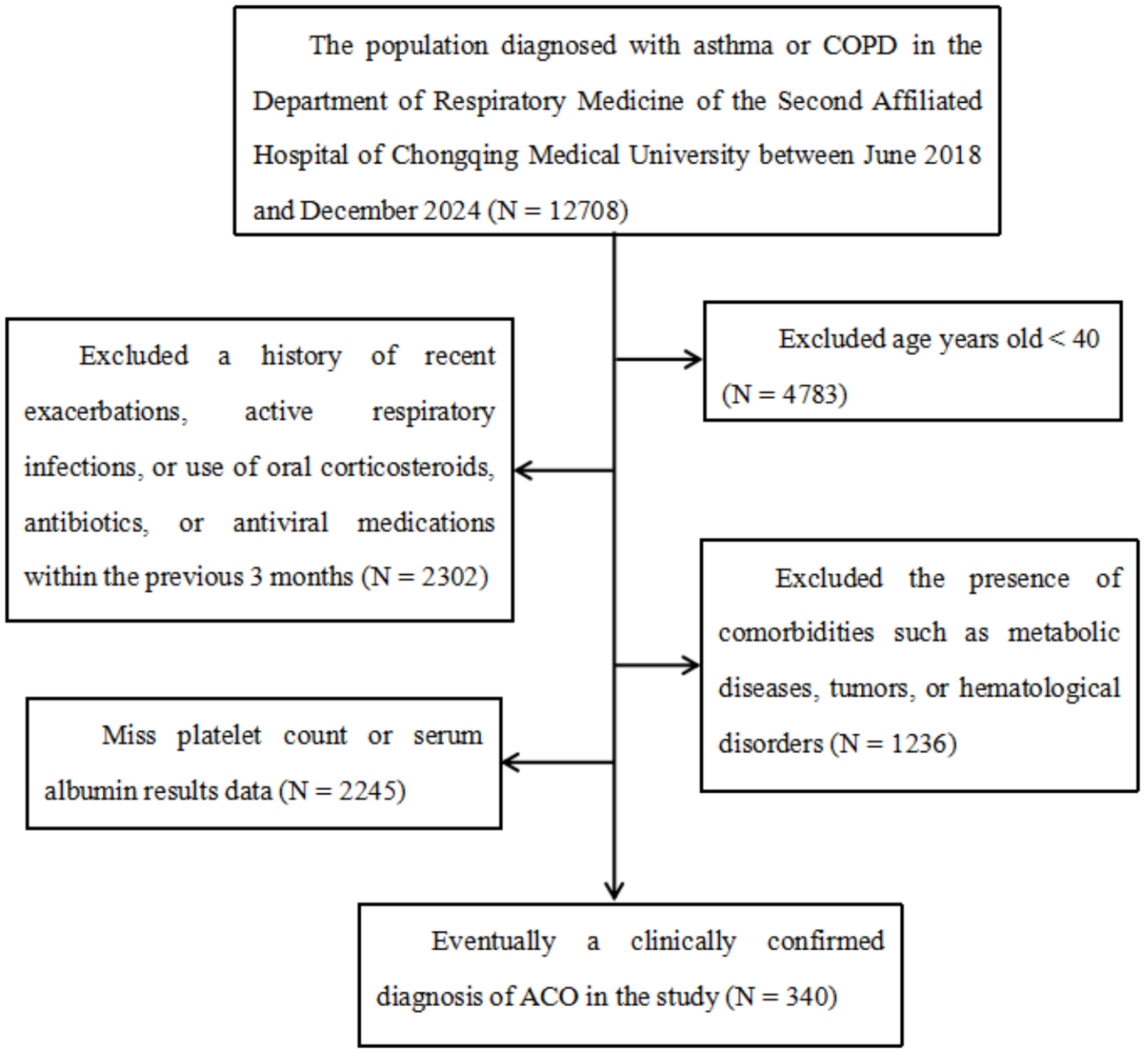
Flowchart illustrating the process of participant selection. COPD, chronic obstructive pulmonary disease; ACO, asthma–chronic obstructive pulmonary disease and asthma overlap.

We conducted a retrospective, single-center study using electronic medical records of patients diagnosed with ACO and healthy controls (HCs) evaluated at the Department of Respiratory Medicine and the Health Examination Center of the Second Affiliated Hospital of Chongqing Medical University between June 2018 and December 2024. The study protocol was approved by the Ethics Committee of the Second Affiliated Hospital of Chongqing Medical University, and written informed consent was obtained from all participants. The inclusion criteria were as follows: (1) age ≥ 40 years, (2) a clinically confirmed diagnosis of ACO, and (3) absence of other underlying pulmonary diseases. The exclusion criteria included: (1) a history of recent exacerbations, active respiratory infections, or use of oral corticosteroids, antibiotics, or antiviral agents within the preceding 3 months; (2) comorbid conditions such as metabolic disorders, malignancies, or hematological diseases; and (3) missing data on platelet counts or serum albumin levels. Detailed inclusion and exclusion criteria have been described previously (22), and the participant screening process is illustrated in Figure 1.

### 2.2 Asthma and COPD diagnosis

Asthma was diagnosed according to the Global Initiative for Asthma (GINA) guidelines (GINA 2025) (23), and COPD was diagnosed based on the Global Initiative for Chronic Obstructive Lung Disease (GOLD 2025) criteria (24).

### 2.3 ACO diagnosis

The major diagnostic criteria for ACO were as follows: (i) persistent airflow limitation, indicated by a post-bronchodilator FEV₁/FVC ratio of < 0.70 in individuals aged ≥ 40 years, (ii) a smoking history of ≥ 10 pack-years, and (iii) either a documented diagnosis of asthma before the age of 40 years or a bronchodilator response (BDR) exceeding 400 mL in FEV₁. The minor criteria included (i) a documented history of atopy or allergic rhinitis, (ii) a BDR of ≥ 200 mL and ≥ 12% in FEV₁ on two or more occasions, and (iii) a peripheral blood eosinophil count of ≥ 300 cells/μL. Patients who fulfilled all major criteria and at least one minor criterion were classified into the ACO cohort (25, 26).

### 2.4 Health participants

Age- and sex-matched health check-up participants were included as controls; they had normal lung function and no underlying medical conditions.

### 2.5 Calculation of PNR index

The PNR was calculated as the ratio of platelet count (×10^9^ cells/L) to neutrophil count (×10^9^ cells/L), using the formula: $\;\mathrm{PNR}=\frac{\mathrm{PLT}(10^{9}/L)}{\mathrm{NP}(10^{9}/L)}$ (19).

### 2.6 Statistical analysis

Categorical variables were compared using the chi-squared test or Fisher’s exact test, as appropriate, while continuous variables were assessed using the Mann–Whitney U test or the Kruskal–Wallis test. Three logistic regression models were constructed: Model I (unadjusted); Model II (adjusted for age and sex); and Model III (adjusted for age, sex, body mass index [BMI], hemoglobin, white blood cell count, eosinophil count, creatinine, alanine aminotransferase, aspartate aminotransferase, albumin, smoking history, and alcohol consumption). Restricted cubic spline (RCS) analyses were performed to evaluate potential non-linear relationships between PNR and ACO, and Spearman’s correlation analysis assessed the strength and direction of associations. Subgroup analyses were conducted to explore potential differences across specific population groups. Receiver operating characteristic (ROC) curve analysis and area under the curve (AUC) values were used to assess the predictive performance of PNR for ACO. Statistical analyses were performed using SPSS version 26 (IBM Corp., Armonk, NY, United States) and R version 4.2.2, and GraphPad Prism version 9.5.1 was used for data visualization. A two-tailed *p*-value of < 0.05 was considered statistically significant.

## 3 Results

### 3.1 Participant baseline characteristics

As shown in Table 1, the study included 1,025 participants, with a median age of 61 years and a median BMI of 24.03 kg/m^2^. Male participants accounted for 72.78% of the cohort, 32.68% were current or former smokers, and 26.24% reported regular alcohol consumption. The median PNR value was 61.55, with quartile distributions as follows: Q1 ≤ 42.19, Q2 = 42.19–57.92, Q3 = 57.92–76.64, and Q4 ≥ 76.64.

**Table 1:** Baseline characteristics of participants.

	Overall (*N* = 1,025)	Q1 ≤ 42.19 (*N* = 257)	42.19 < Q2 ≤ 57.92 (*N* = 257)	57.92 < Q3 ≤ 76.64 (*N* = 255)	Q4 ≥ 76.64 (*N* = 256)	*p*-value
Age(years)	61.00 (40.00–94.00)	63.00 (40.00–94.00)	61.00 (40.00–91.00)	60.00 (41.00–81.00)	59.00 (40.00–84.00)	< 0.001
BMI(kg/m^2^)	24.03 (15.37–47.65)	24.09 (15.37–37.44)	24.16 (16.16–35.55)	24.20 (17.19–47.65)	23.83 (16.79–33.77)	0.196
White blood cells(10^9^/L)	6.11 (2.49–29.51)	8.56 (4.07–29.51)	6.53 (2.66–11.88)	5.83 (2.88–9.57)	4.84 (2.49–9.62)	<0.001
Hemoglobin(g/L)	142.00 (64.00–201.00)	142.00 (64.00–201.00)	144.00 (82.00–175.00)	144.00 (95.00–185.00)	137.00 (94.00–172.00)	<0.001
Neutrophil(10^9^/L)	3.62 (1.19–28.56)	5.93 (2.43–28.56)	4.00 (1.85–9.55)	3.39 (2.01–6.27)	2.51 (1.19–6.09)	<0.001
Neutrophil(%)	60.10 (19.90–98.60)	72.60 (19.90–98.60)	62.40 (44.40–93.70)	57.70 (40.70–71.00)	52.45 (30.90–76.60)	<0.001
Lymphocytes(%)	29.20 (0.50–135.00)	17.80 (0.50–85.90)	27.10 (0.57–135.00)	31.50 (13.50–51.00)	36.40 (5.10–60.40)	<0.001
Eosinophil(10^9^/L)	0.14 (0.00–4.17)	0.10 (0.00–4.17)	0.13 (0.00–2.64)	0.15 (0.00–1.02)	0.16 (0.01–1.78)	<0.001
Platelets(10^9^/L)	216.00 (53.00–631.00)	188.00 (53.00–407.00)	202.00 (88.00–522.00)	224.00 (129.00–450.00)	244.50 (137.00–631.00)	<0.001
Creatinine(mg/dl)	71.80 (6.50–217.60)	72.40 (24.80–176.90)	72.00 (36.30–125.90)	74.30 (6.50–217.60)	66.90 (41.70–128.50)	<0.001
Alanine aminotransferase(IU/L)	21.00 (2.00–183.00)	20.00 (2.00–149.00)	21.00 (7.00–183.00)	23.00 (7.00–93.00)	20.00 (6.00–180.00)	0.008
Aspartate aminotransferase(IU/L)	22.00 (8.00–195.00)	21.00 (10.00–130.00)	22.00 (8.00–195.00)	23.00 (11.00–70.00)	23.00 (12.00–83.00)	0.002
Albumin(g/L)	45.40 (37.10–53.70)	45.42 (37.70–51.80)	45.40 (37.50–53.60)	45.45 (40.00–53.00)	45.40 (37.10–53.70)	0.633
Sex(%)						0.288
Male	746(72.78%)	183(71.21%)	178(69.26%)	194(76.08%)	191(74.61%)	
Female	279(27.22%)	74(28.79%)	79(30.74%)	61(23.92%)	65(25.39%)	
Smoking history(%)						<0.001
No	690(67.32%)	129(50.19%)	161(62.65%)	183(71.76%)	217(84.77%)	
Yes	335(32.68%)	128(49.81%)	96(37.35%)	72(28.24%)	39(15.23%)	
Drinking history(%)						0.012
No	756(73.76%)	182(70.82%)	182(70.82%)	183(71.76%)	209(81.64%)	
Yes	269(26.24%)	75(29.18%)	75(29.18%)	72(28.24%)	47(18.36%)	
ACO(%)						<0.001
No	685(66.83%)	81(31.52%)	167(64.98%)	218(85.49%)	219(85.55%)	
Yes	340(33.17%)	176(68.48%)	90(35.02%)	37(14.51%)	37(14.45%)	

For categorical variables, Fisher’s exact test and the chi-squared statistic test were utilized, while the Mann–Whitney U test and T-test were used for continuous variables.

### 3.2 Compare the PNR in ACO patients and HCs

**Figure 2:**
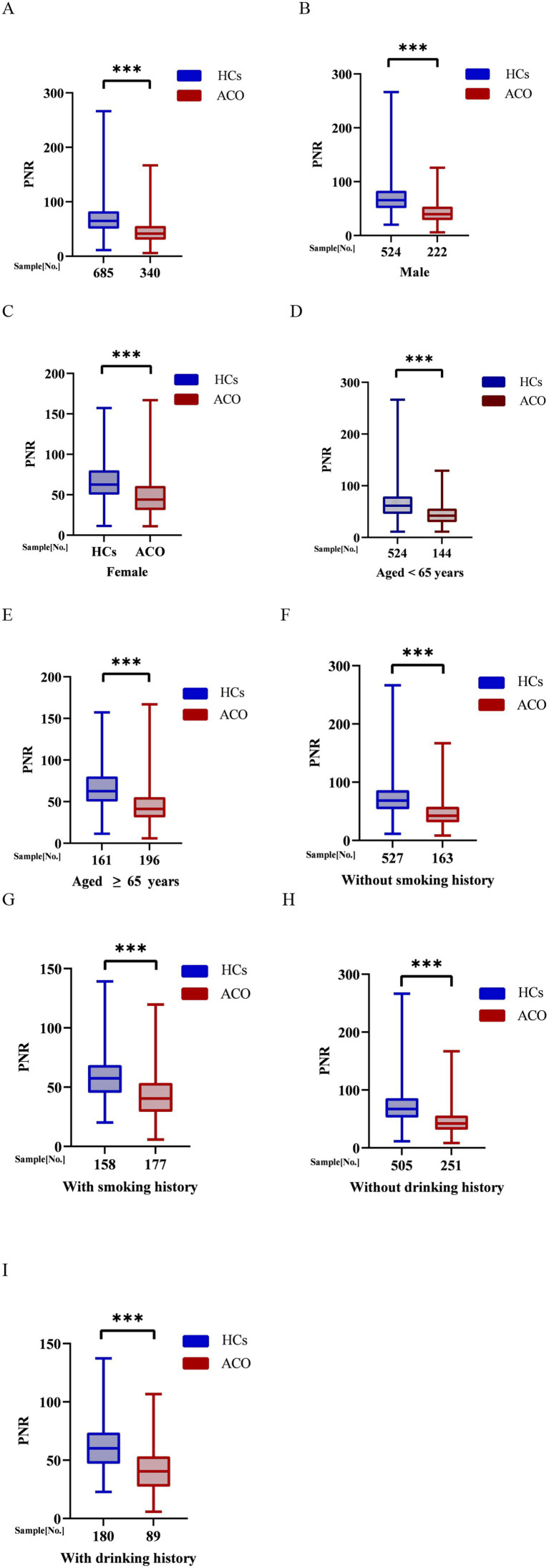
PNR in ACO patients and HCs. **(A)** all; **(B)** male; **(C)** female; **(D)** aged < 65 years; **(E)** aged ≥ 65 years; **(F)** without smoking history; **(G)** with smoking history; **(H)** without drinking history; **(I)** with drinking history. PNR, platelets-to-neutrophil ratio; ACO, asthma–chronic obstructive pulmonary disease overlap.

Compared with the ACO group, PNR values were significantly higher in HCs (*p* < 0.001; Figure 2A). This difference remained significant in both sexes, with male and female HCs exhibiting higher PNR levels than their ACO counterparts (*p* < 0.001; Figures 2B,C). Similarly, PNR was significantly elevated in HCs compared with ACO patients in both age subgroups—those aged ≥ 65 years and those aged < 65 years (Figures 2D,E). This pattern persisted regardless of smoking status, with HCs showing higher PNR levels than ACO participants among both smokers and non-smokers (*p* < 0.001; Figures 2F,G). PNR also remained significantly higher in HCs than in ACO patients, irrespective of alcohol consumption (*p* < 0.001; Figures 2H,I).

### 3.3 Analysis of the correlation between PNR index and ACO

After adjusting for all relevant covariates, higher PNR was significantly associated with a reduced risk of ACO (OR = 0.964; 95% CI: 0.954–0.975; *p* < 0.0001). Compared with participants in the lowest quartile (Q1), those in quartiles Q2, Q3, and Q4 demonstrated progressively lower odds of ACO. In the fully adjusted model (Model 3), the odds ratios were 0.345 (95% CI: 0.211–0.565) for Q2, 0.126 (95% CI: 0.071–0.223) for Q3, and 0.126 (95% CI: 0.065–0.241) for Q4, with a significant trend observed across quartiles (P for trend < 0.0001; Table 2). These associations were consistent across all regression models.

**Table 2:** Multivariable logistic regression models examining the association between PNR and ACO.

Variable	Model 1		Model 2		Model 3	
	OR (95% CI)	*p*	OR (95% CI)	*p*	OR (95% CI)	*p*
PNR	0.956 (0.949,0.963)	< 0.0001	0.958 (0.950,0.965)	< 0.0001	0.964 (0.954,0.975)	< 0.0001
PNR (Q1)	Reference		Reference		Reference	
PNR (Q2)	0.245 (0.170,0.354)	< 0.0001	0.247 (0.169,0.361)	< 0.0001	0.345 (0.211, 0.565)	< 0.0001
PNR (Q3)	0.080 (0.052,0.124)	< 0.0001	0.087 (0.056,0.135)	< 0.0001	0.126 (0.071, 0.223)	< 0.0001
PNR (Q4)	0.078 (0.050,0.120)	< 0.0001	0.088 (0.056,0.137)	< 0.0001	0.126 (0.065, 0.241)	< 0.0001
P for trend		< 0.0001		< 0.0001		< 0.0001

Model I featured no adjustment for covariates, whereas Model II incorporated adjustments for age and sex. Moving on to Model III, a comprehensive adjustment was made for age, sex, BMI, hemoglobin, white blood cells, eosinophils, creatinine, alanine aminotransferase, aspartate aminotransferase, albumin, smoking history, and drinking history. PNR, platelet-to-neutrophil ratio; OR, odds ratio; CIs, confidence intervals.

### 3.4 Analysis of smooth fitting curve and threshold effects

**Figure 3:**
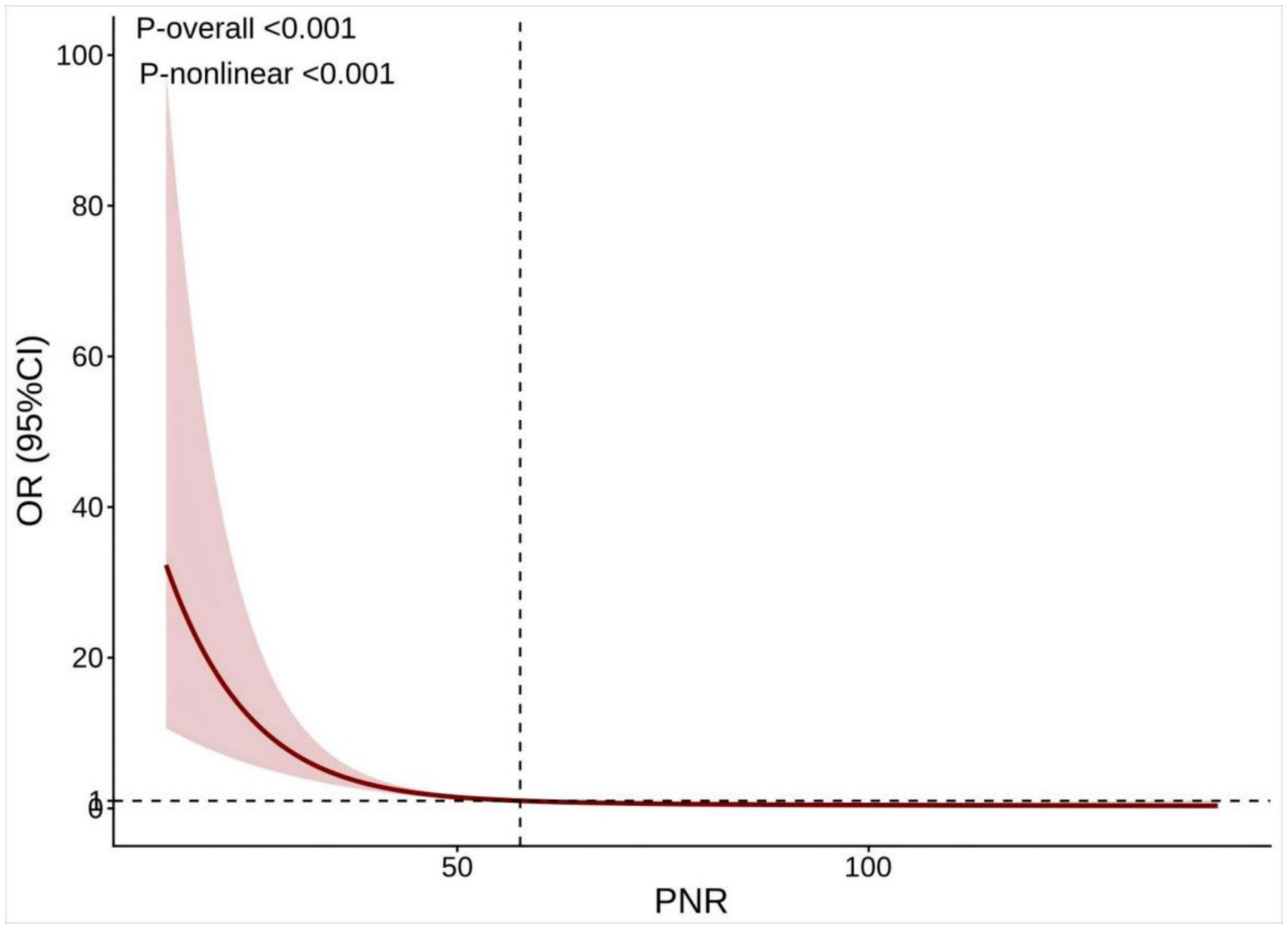
Restricted cubic spline between PNR and ACO. The pink bars show the fitted 95% confidence intervals (95% CI), and the fitted curves are shown in red. PNR, platelet-to-neutrophil ratio; ACO, asthma–chronic obstructive pulmonary disease overlap.

The smooth fitting curve in Figure 3 demonstrates a non-linear relationship between PNR and ACO. Threshold effect analysis identified an inflection point at a PNR value of 61.17. Below this threshold, higher PNR was significantly associated with a reduced risk of ACO (OR = 0.926; 95% CI: 0.905–0.948; *p* < 0.0001). In contrast, when PNR exceeded 61.17, the association was no longer statistically significant (OR = 0.992; 95% CI: 0.976–1.007; *p* = 0.302; Table 3). The likelihood ratio test confirmed the presence of this non-linear relationship (*p* < 0.0001).

**Table 3:** Threshold effect analysis of the PNR on ACO risk.

	PNR The effect size (95%CI)	*p*-value
Model 1: Fitting model by standard linear regression	0.964(0.954, 0.975)	< 0.0001
Model 2: Fitting model by two-piecewise linear regression		
Inflection point(K)	61.17	
≤ K	0.926(0.905, 0.948)	< 0.0001
> K	0.992(0.976, 1.007)	0.302
P for likelihood ratio test		< 0.0001

PNR, platelet-to-neutrophil ratio; OR, odds ratio; CI, confidence intervals.

### 3.5 Subgroup analyses

**Figure 4:**
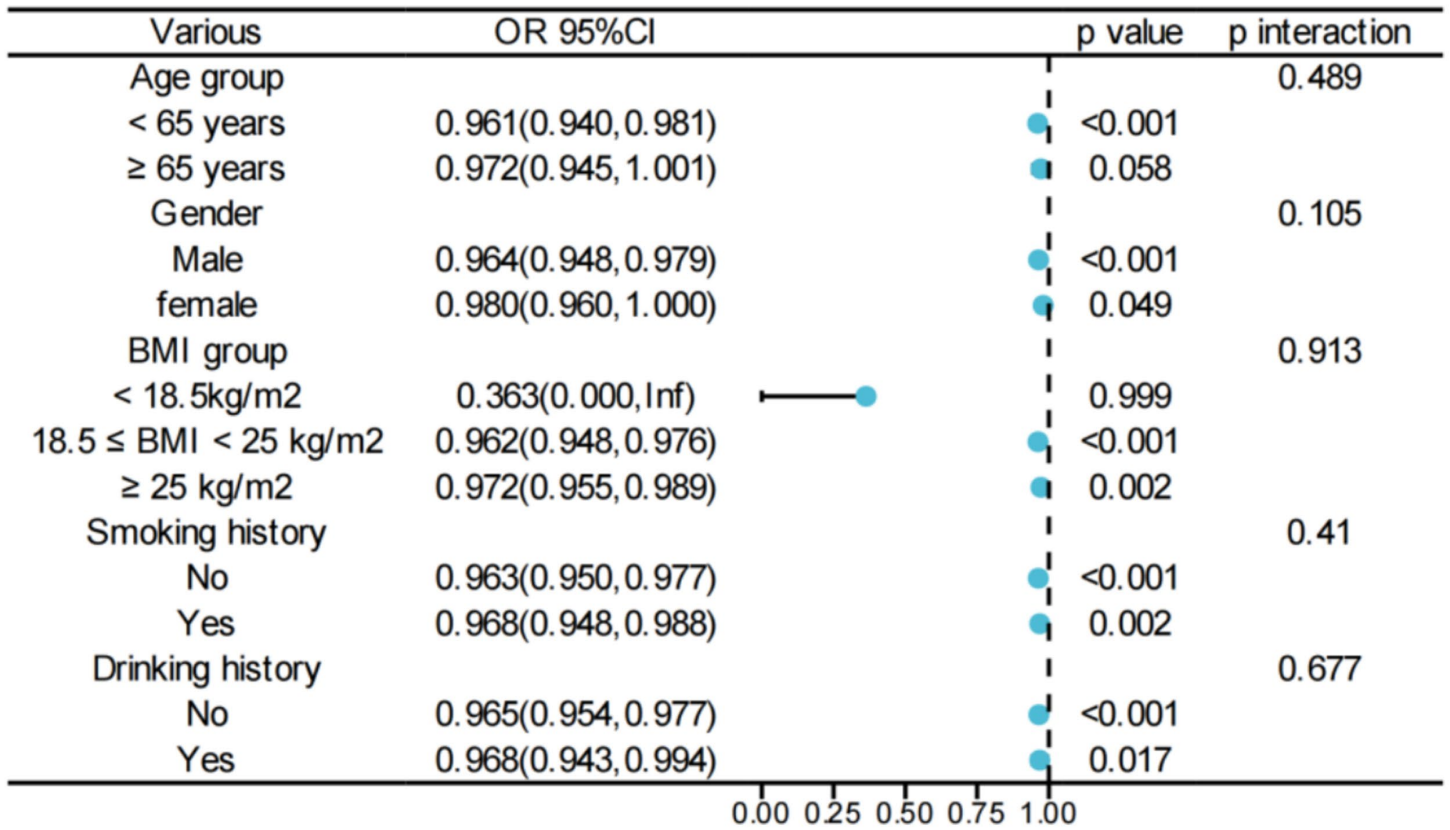
Subgroup analysis of the PNR–ACO correlation. BMI, body mass index; OR, odds ratio; CIs, confidence intervals.

Comprehensive subgroup analyses and interaction tests, adjusted for all relevant covariates, were performed to evaluate the robustness of the association between PNR and ACO and to examine potential variations across different populations. A consistent and significant inverse relationship between PNR and ACO was observed across most subgroups. Interaction analyses, however, indicated no statistically significant differences in the PNR–ACO association among subgroups (all *p* > 0.05; Figure 4).

### 3.6 The correlation analysis between PNR and COPD risk

**Figure 5:**
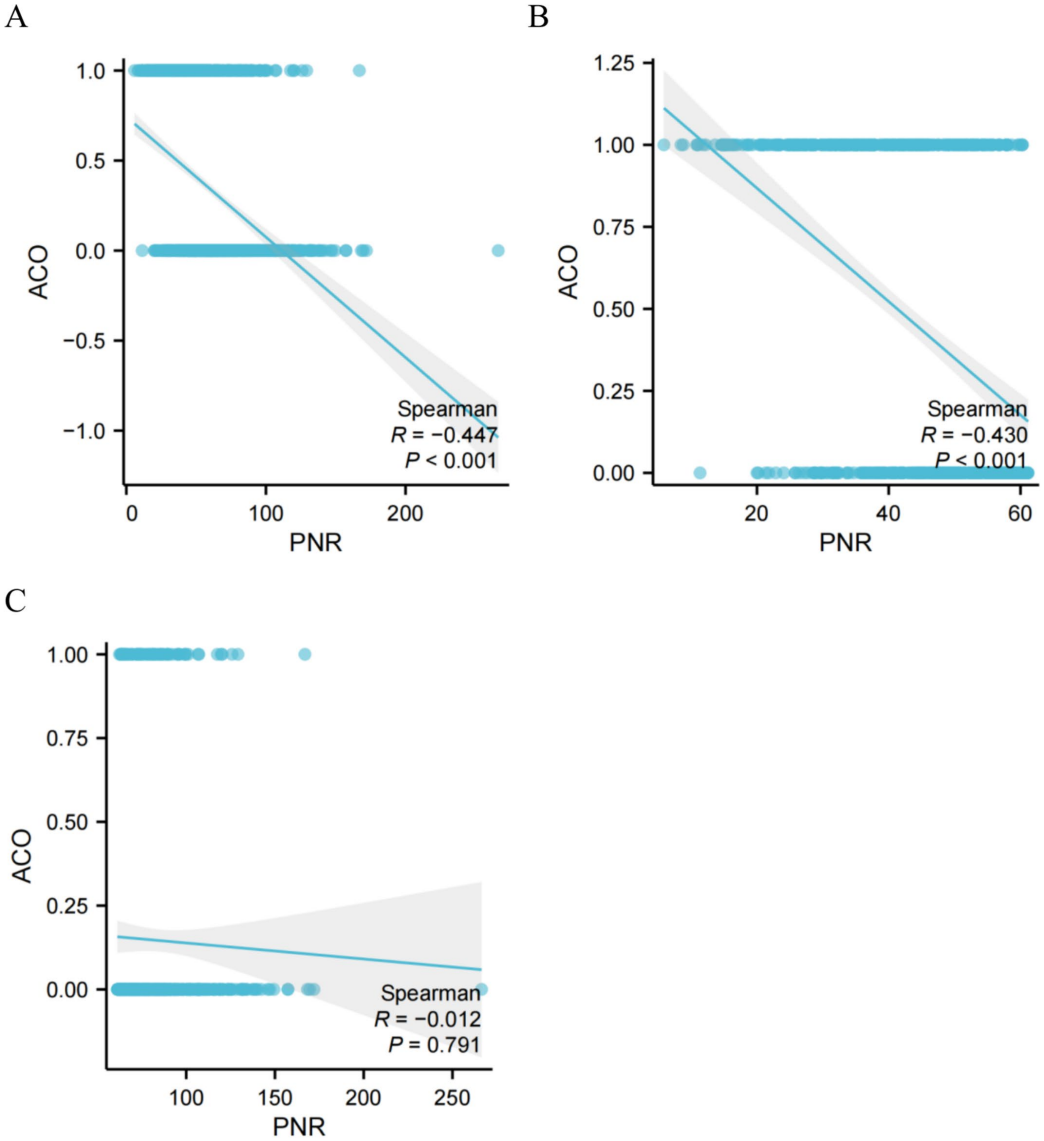
Correlation analysis between PNR and COPD risk. **(A)** All PNR, **(B)** PNR < 61.17, and **(C)** PNR ≥ 61.17. PNR, platelet-to-neutrophil ratio; ACO, chronic obstructive pulmonary disease and asthma overlap.

Spearman’s correlation analysis revealed a significant negative correlation between PNR and ACO overall (*r* = −0.447, *p* < 0.001), which remained significant after adjusting for relevant covariates (Figure 5A). This inverse relationship was particularly pronounced when PNR was below the threshold of 61.17 (*r* = −0.430, *p* < 0.001; Figure 5B). In contrast, no significant correlation was observed when PNR was ≥ 61.17 (*r* = −0.012, *p* = 0.791; Figure 5C).

### 3.7 Predict ACO risk

**Figure 6:**
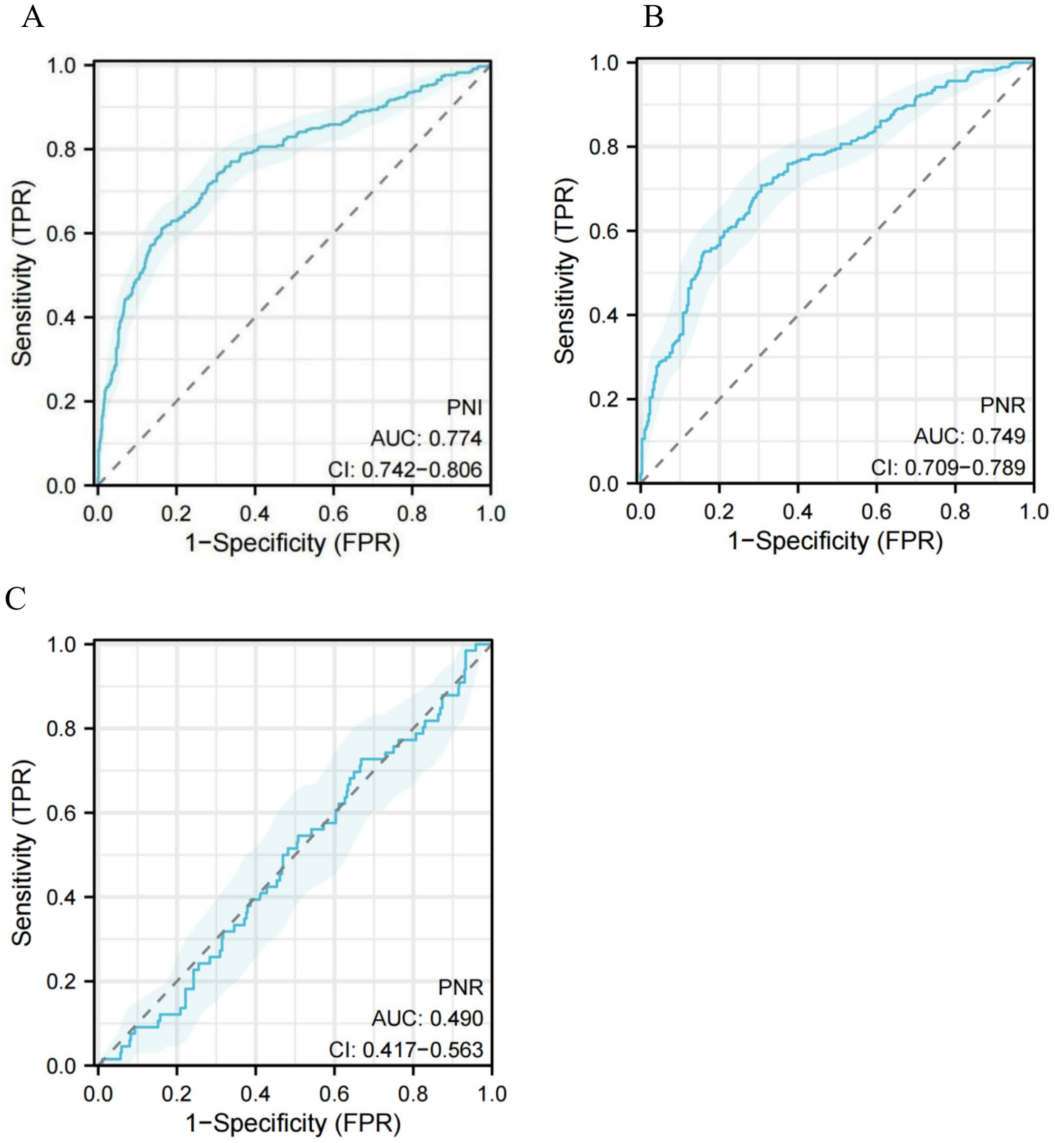
ROC to assess the ACO risk. **(A)** All PNR, **(B)** PNR < 61.17, and **(C)** PNR ≥ 61.17. PNR, platelets-to-neutrophil ratio; ROC, receiver operating characteristic curve; AUC, area under the curve; CIs, confidence intervals.

ROC curve analysis was performed to assess the predictive value of PNR for ACO risk. The overall AUC was 0.774 (95% CI: 0.742–0.806; Figure 6A), with the calibration curve shown in Supplementary Figure 1. Using a cutoff value of 46.149, the sensitivity was 0.612, the specificity was 0.837, and the Youden index was 0.749 (Supplementary Table 1). When stratified by the threshold of PNR < 61.17, the AUC was 0.749 (95% CI: 0.709–0.789; Figure 6B). In contrast, for PNR ≥ 61.17, predictive performance declined markedly, with an AUC of 0.490 (95% CI: 0.417–0.563; Figure 6C).

## 4 Discussion

In this study, we found that patients with ACO exhibited significantly lower PNR levels compared with healthy controls. Furthermore, our analyses revealed a non-linear association and threshold effect between PNR and ACO, a relationship that, to our knowledge, has not been previously reported, underscoring the potential role of PNR as an inflammatory biomarker in the context of ACO.

Platelets are small, anucleate cellular fragments derived from megakaryocytes, with approximately one trillion circulating in the adult bloodstream (27, 28). Beyond their classical roles in hemostasis and thrombosis, platelets are increasingly recognized as immune cells that participate in diverse immunological processes (29, 56). Numerous studies have documented platelet recruitment and localization within pulmonary tissues, where platelets contribute to inflammatory responses through interactions with dendritic cells, eosinophils, and neutrophils, thereby modulating immune function (30–32). Platelets also secrete a variety of bioactive mediators, including growth factors (33–35), adenosine triphosphate (36), histamine (37), serotonin (38), interleukin-33 (39), platelet-activating factor (40, 41), and thromboxane A2 (42), which can exacerbate airway inflammation. Neutrophilic inflammation is a hallmark of both healthy lungs and various acute and chronic pulmonary diseases, including acute bronchitis, acute respiratory distress syndrome, COPD, and severe asthma. Neutrophils are recruited to inflammatory sites by chemotactic factors, including IL-8, leukotriene B4, and tumor necrosis factor-alpha, and can cause tissue damage through excessive activation and the release of reactive oxygen species (43, 44). In COPD, the aberrant inflammatory response involves neutrophils, macrophages, and CD8 + T lymphocytes, leading to airway remodeling and functional impairment. Emphysema is characterized by disruption of the protease–antiprotease balance mediated by neutrophils, resulting in alveolar destruction and impaired mucociliary clearance. Chronic bronchitis involves mucosal infiltration by neutrophils, macrophages, and lymphocytes, causing epithelial damage, smooth muscle hypertrophy, and fibrosis (43, 44).

The PNR is an emerging biomarker that integrates platelet and neutrophil counts, providing a comprehensive reflection of both thrombotic and inflammatory status, as well as their interactions. Previous studies have demonstrated the clinical utility of PNR across diverse conditions. For example, PNR exhibits superior prognostic accuracy compared with PLR and the platelet-to-white blood cell ratio in predicting 3-month outcomes after acute ischemic cerebral infarction (18). It also outperforms NLR and PLR in distinguishing patients with preeclampsia and gestational hypertension (45) and serves as a better predictor of impending brain death than PLR (46). In cerebrovascular diseases, lower PNR values are generally associated with poorer prognosis and an increased risk of hemorrhagic transformation in acute ischemic stroke (47). Abounoori et al. (48) reported progressive declines in PNR in patients with Graves’ ophthalmopathy, correlating with disease severity. Similarly, patients with diabetic macular edema exhibit significantly lower PNR levels compared with HCs (10), findings that are consistent with those of the present study.

In ACO, persistent inflammation plays a central role in platelet activation and functional modulation. Chronic hypoxia, a hallmark of ACO, stimulates megakaryocytes in the bone marrow, affecting platelet production and morphology, and leading to variations in platelet count and size (49). Compared with patients with isolated asthma or COPD, individuals with overlapping features tend to exhibit altered platelet dynamics and greater susceptibility to stress-induced platelet changes (50). Furthermore, both ACO and asthma patients show reduced neutrophil numbers in the airway mucosa (51). Previous studies have reported that ACO patients present with elevated eosinophil counts but reduced neutrophil percentages, alongside decreased NLR and PLR values (52). Notably, patients with COPD or asthma generally exhibit elevated inflammatory markers, including PLR, NLR, monocyte-to-lymphocyte ratio, basophil-to-lymphocyte ratio, and eosinophil-to-lymphocyte ratio (53–55). Our findings indicate that PNR is lower in ACO patients compared with HCs and is inversely correlated with ACO. This finding may reflect persistent chronic inflammatory responses that influence platelet production and function. Under inflammatory conditions, increased neutrophil production can further reduce PNR. Additionally, airway remodeling and impaired pulmonary function in ACO may contribute to decreased PNR through complex regulatory mechanisms involving inflammatory mediators in the hematopoietic and immune systems, highlighting the intricate interplay between inflammation, platelet behavior, and the unique immunological profile of ACO. A reduction in PNR may also signal a predisposition to “immune thrombosis,” in which activated platelets interact with neutrophils to form platelet–neutrophil aggregates, leading to microvascular embolism and exacerbating local hypoxia and inflammation. This interaction can further activate additional immune cells, releasing pro-inflammatory mediators and creating a positive feedback loop that perpetuates the inflammatory response.

This study has several limitations. First, as a single-center, cross-sectional, retrospective study, it is susceptible to selection bias, which may affect the robustness of our conclusions. Prospective, randomized controlled studies with serial measurements are needed to establish causality and strengthen the evidence base. Second, although we adjusted for numerous potential confounders, the possibility of residual confounding cannot be excluded. Third, the relative homogeneity of the study population limits the generalizability of our findings to other racial and ethnic groups. Finally, potential errors in data collection may have introduced bias. Despite these limitations, our study provides novel insights into the role of PNR as an inflammatory biomarker in ACO and offers important directions for future research.

## 5 Conclusion

This study demonstrated a significant inverse association between PNR and ACO, particularly when PNR is below 61.17. These findings suggest that PNR may serve as a promising biomarker for ACO, with potential applications in risk stratification and patient management. Future research should aim to elucidate the mechanistic links between PNR and ACO pathogenesis and to validate its clinical utility across diverse populations. Larger studies are also warranted to compare the diagnostic performance of PNR in patients with asthma-only, COPD-only, and overlapping phenotypes.

## Data Availability

The original contributions presented in the study are included in the article/Supplementary material, further inquiries can be directed to the corresponding author.
