# Supplementary figures and images for: Association between platelet-to-neutrophil ratio and asthma–COPD overlap: a cross-sectional study in China
Source: Front Med (Lausanne). 2026 Mar 18;13:1729278. doi: 10.3389/fmed.2026.1729278 (PMC13039030; doi:10.3389/fmed.2026.1729278)

| A B  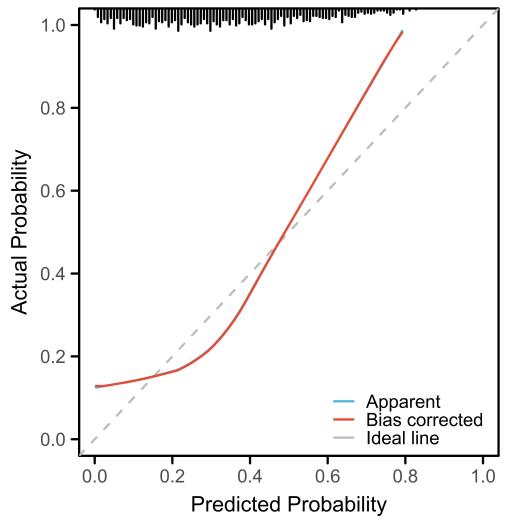 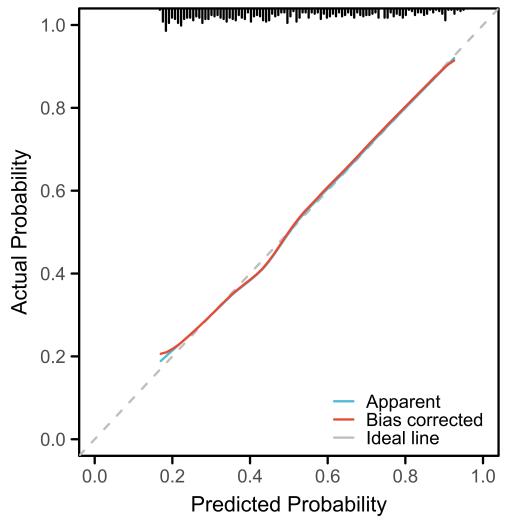  C  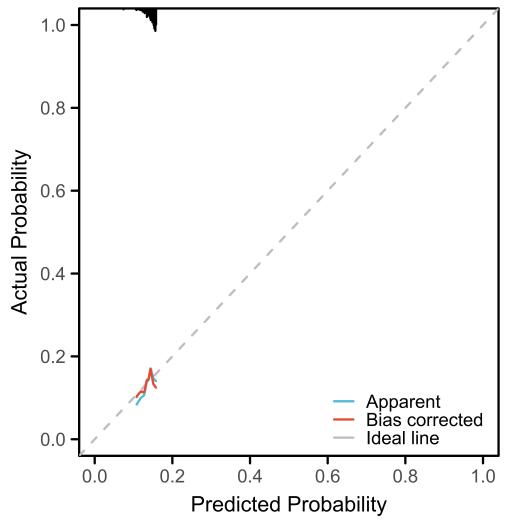  Supplementary Figure 1. The calibration curve between PNR and COPD risk. (A) all PNR; (B) PNR < 61.17; (C) PNR ≥ 61.17. |
| --- |

Supplement: Supplementary file 1 [file Supplementary_file_1.docx]
