# Supplementary material for: Association between platelet-to-neutrophil ratio and asthma–COPD overlap: a cross-sectional study in China
Source: Front Med (Lausanne). 2026 Mar 18;13:1729278. doi: 10.3389/fmed.2026.1729278 (PMC13039030; doi:10.3389/fmed.2026.1729278)
Supplement: Supplementary file 2 [file Table_1.docx]

| **PNR** | **Cutoff** | **Sensitivity** | **Specificity** | **Precision** | **Positive rate** | **Negative rate** | **Youden index** |
| --- | --- | --- | --- | --- | --- | --- | --- |
| All | 46.149 | 0.612 | 0.838 | 0.763 | 0.652 | 0.813 | 0.749 |
| < 61.17 | 44.421 | 0.708 | 0.694 | 0.701 | 0.681 | 0.72 | 0.752 |
| ≥ 61.17 | 72.026 | 0.727 | 0.332 | 0.39 | 0.156 | 0.878 | 0.059 |

Supplementary table 1.The predict relevant indicators between PNR and ACO. PNR, Platelets to Neutrophil Ratio; ACO, Chronic Obstructive Pulmonary Disease and Asthma Overlap.
